# Knowledge and Attitudes of Nursing Students Regarding Children’s Pain

**DOI:** 10.7759/cureus.69321

**Published:** 2024-09-13

**Authors:** Hawa M Alabdulaziz, Salmah Alghamdi, Sarah M Alabbadi, Rahaf J Halawani, Razan A Alsulami, Sarah H Hakami

**Affiliations:** 1 Department of Maternity and Child Nursing, King Abdulaziz University, Jeddah, SAU; 2 Department of Medicine and Surgery, King Faisal Specialist Hospital and Research Centre, Jeddah, SAU; 3 Department of Obstetrics and Gynecology, King Faisal Specialist Hospital and Research Centre, Jeddah, SAU; 4 Department of Medicine and Surgery, Ministry of Health, Jeddah, SAU

**Keywords:** attitude, children’s pain, knowledge, nursing students, pain management

## Abstract

Background: Although the occurrence of pain among hospitalized children is inevitable, the way to assess and detect the intensity of pain is still challenging to pediatric professionals and may lead to increased morbidity and mortality rates if left untreated. The knowledge base of nurses regarding both pharmacological and non-pharmacological pain management among children plays a significant role in managing and making a management plan. The purpose of this study was to assess nursing students’ knowledge and attitudes toward pediatric pain management.

Methods: This study used a cross-sectional design to investigate the knowledge and attitudes regarding pain and its management among undergraduate nursing students at a university. A convenience sample of students from all levels was obtained. The pediatric nurses knowledge and attitudes survey (PNKAS) regarding pain was used. Data were analyzed by IBM SPSS Statistics for Windows, Version 22 (Released 2013; IBM Corp., Armonk, New York, USA).

Results: In this study, 86.3% of participants (n = 69) scored within the satisfactory range on the PNKAS, suggesting adequate knowledge and attitudes about pediatric pain management. The results showed there was no signiﬁcant relationship between age and a total score p-value of 0.740, as well as between academic levels and the same p-value of 0.397.

Conclusion: This study highlights the importance of undergraduate nursing education in pediatric pain management. The findings suggest that students may benefit from additional training in pain assessment and pharmacological interventions.

## Introduction

Pain is defined as an unpleasant feeling and emotional expertise related to actual or potential harm [1]. The cognitive, emotional, as well as behavioral elements in pediatric care are also necessary in pain assessment to clarify the management practices [2]. Moreover, uncontrolled pain has an additional direct effect on patients' health outcomes [3].

Assessment and management of pain are necessary elements of pediatric care. Compared to adults, it is more challenging to assess and manage pain successfully in the pediatric population [3]. Therefore, nurses have to educate patient’s families about pain management and ensure that all treatments have been delivered [4].

Pediatric pain management requires understanding a child's pain expressions for proper assessment and intervention [5]. Inaccurate assessment can leave children vulnerable to recurrent and procedural pain [3].

Unlike adults, pediatric pain is influenced by developmental factors that impact emotional processing, pain perception, and treatment response throughout childhood and adolescence [5,6]. This necessitates a focus on pain management education for nurses, who play a crucial role in assessing and managing children's pain [7]. Well-equipped nurses can significantly alleviate suffering through effective pain assessment and management techniques.

Pain assessment, considered the fifth vital sign, is crucial for understanding a patient's pain characteristics and guiding optimal management plans. This empowers nurses to deliver appropriate interventions and improve patient care. However, pediatric pain assessment presents unique challenges due to communication difficulties and limitations in self-expression.

A study conducted in Hail used a cross-sectional descriptive design to assess knowledge and attitudes regarding pain management [8]. The study included a sample of 150 undergraduate Saudi nursing students at the University of Hail in Saudi Arabia. A Survey Regarding Pain Scale was used. Data was collected over a two-month period from April 2017 through June 2017. The data revealed a lack of knowledge and attitudes toward pain management among nursing students and recommended that additional training education should be addressed.

Another study was conducted in Taiwan to compare students' skills and knowledge following the researcher building a program about pain management by using three teaching strategies: e-learning, traditional face-to-face, or blended learning [9]. The sample involved 296 undergraduate nursing students in Taiwan. The study's aims were to support the existing pediatric pain management education and to test the beneficence of administering the program strategies. The pediatric pain management program included the meaning of pain, pharmacological and non-pharmacological pain interventions, misconceptions about children's pain, and a child’s pain nursing care plan. The results revealed that there was no difference between all three groups.

Another study conducted in Riyadh used a cross-sectional descriptive design to test the knowledge and attitudes of pediatric nurses [10]. The study included a sample of 410 registered pediatric nurses working at five Saudi Arabian government hospitals. The study used the pediatric nurses knowledge and attitudes survey regarding pain (PNKAS-Shriners Revision). The study demonstrated that there was a lack of knowledge and attitudes regarding pediatric pain management. The study recommended pediatric pain intensive programs and their management.

A study was conducted in Ghana using a comparative cross-sectional study to describe nursing students' and nurses' attitudes and levels of knowledge regarding pediatric pain [11]. The total participant was 65 nurses and 554 final-year nursing students. The results showed that both groups had deficient knowledge and attitudes toward pain management in children. Moreover, nursing students had higher scores in total PINKS in 10 out of 13. They showed the need to enforce educational programs in this area.

A study was conducted at Connecticut University in the United States using a cross-sectional study to describe nursing students' attitudes and levels of knowledge regarding pediatric pain [12]. The total participants were 135 junior and 112 senior nursing students. The results showed that the majority of students had difficulty with questions relating to pharmacology in pediatric pain management; however, juniors performed better than fourth- and fifth-year seniors. The study recommends providing curriculum revision and educational interventions to increase sensitivity and competence in caring for pediatric patients with pain.

Moreover, a study conducted in Jordan used a cross-sectional study to test the nursing students' knowledge and attitudes toward children’s pain management [13]. The total number of participants was 101 nursing students. They found the students lacked knowledge and attitudes toward pediatric pain assessment and recommended more educational materials throughout their study plans.

A study was done in Egypt using a descriptive cross-sectional to evaluate the knowledge and attitudes of undergraduate nursing students about the management of pain in children [14]. The total participants were 471 third- and fourth-year undergraduate nursing students. The study used nurses’ knowledge and attitudes survey regarding pain (PNKAS). They found that nursing students lack pain management skills and attitudes when using (PNKAS), particularly in pain assessment and pharmacological pain management. The study suggested the training and management of new graduate nursing students with theoretical knowledge of pain in clinical practice and a focus on pharmacological and non-pharmacological treatment.

A study was conducted in North Carolina using a cross-sectional quantitative study to examine the relationship between pain management skills and self-efficacy of pediatric nurses [15]. The study used two tools (PNKAS) and nurse self-effectiveness in controlling children’s pain. The result showed no connection between self-efficacy level and knowledge level. The results suggested that nurses need more educational programs about pain management in pediatric patients.

Another study was conducted in Mississippi using a pilot descriptive qualitative study to investigate the perception of nurses regarding pain in pediatric patients [16]. They found that it's hard to assess pain. Another study done in Mongolia aims to evaluate current knowledge of pediatric pain and to determine the efficacy of educational intervention in enhancing pediatric nursing knowledge and attitudes of pediatric nurses [17]. Magnolia nurses’ knowledge and attitudes were evaluated before and after two hours of educational intervention. A total of 155 nurses completed the pre- and post-surveys. The results showed that the educational intervention in Mongolian nurses was successful in improving awareness and attitudes about pediatric pain.

In summary, from the above-mentioned studies, it was shown that there is a lack of appropriate knowledge and attitudes regarding pediatric pain and its management. It is recommended that additional training and education be addressed. In addition, it was evident that most of the research was conducted in areas other than the Middle East. Thus, it is crucial to have more studies to assess nursing students’ knowledge and attitudes toward a child’s pain. This study aims to assess undergraduate nursing students’ knowledge and attitudes toward pediatric pain management at a University in Jeddah.

## Materials and methods

Research design

A quantitative descriptive cross-sectional study was carried out to investigate the knowledge and attitudes regarding pain and its management among undergraduate nursing students at a University in Jeddah.

Sample and sample size

All of the undergraduate nursing students in the Faculty of Nursing at King Abdulaziz University in Jeddah, Saudi Arabia, constituted the study population who were enrolled and are willing to participate in this study. The selection of participants was done through convenience sampling techniques to come up with a representative sample for each level. A total of 80 undergraduate students who agreed to participate in the current study constituted the study sample, distributed as follows: 42.5% from the second academic level, 28.7% from the third academic level, and 28.7% from the fourth academic level.

Tool

A PNKAS-Shirners Revision was used to collect the data. The tool was developed by Manworren (2000) to assess nurses, staff, and nursing students' level of knowledge and attitudes about pediatric pain in different languages [18]. The questionnaire was available online; also, the author's permission was obtained. The questionnaire consists of three sections as follows: 23 true and false questions, 13 multiple-choice questions, and the presentation of two clinical cases.

Each question is assigned a score of 1 for a correct answer and 0 for an incorrect answer. The total score is determined by summing the individual scores for each question. Total scores exceeding 50% on the PNKAS may indicate a satisfactory level of knowledge and attitudes regarding pediatric pain management. Conversely, scores below 50% may indicate an unsatisfactory level of knowledge and attitudes regarding pediatric pain management.

Data collection procedure

The research team has converted the questionnaire into a Google Form to create the uniform resource locator (URL) link for participation. The URL of an electronic Google survey was sent to all nursing students through social media (second, third, and fourth year). Participation was entirely voluntary. The participants acknowledge that they have read and understood the informed consent form and agree to participate in the survey by clicking "Agree." Clicking "Disagree" will exit the survey.

Data analysis

The data was analyzed by IBM SPSS Statistics for Windows, Version 22 (Released 2013; IBM Corp., Armonk, New York, USA). Descriptive statistics such as mean, standard deviation, percentages, and frequency variables were calculated. Inferential statistics are conducted by using the independent sample t-test and one-way ANOVA test. Statically, significance was set at a p-value <0.05.

Ethical considerations

Ethical approval was obtained from the Ethics and Research Committee, Faculty of Nursing at King Abdulaziz University (No. 2F.30), on March 5, 2020. Data collection was started on March 8, 2020, and ended on April 25, 2020. The participants' rights were respected and protected; we aimed to protect the participants' information and confidentiality, and all participant anonymity was protected. Approval for using this questionnaire was obtained from the author. An information sheet was developed for participants to know more about the study.

## Results

The total number of nursing students who participated in this study was 80. Table 1 describes the participants’ demographic characteristics.

**Table 1:** Demographic characteristics of participants The data has been presented as frequency N, (%)

Item		Count (N)	Percentage (%)
Academic level	Second year	34	42.5%
	Third year	23	28.75%
	Fourth year	23	28.75%
Age	Less than 20 years	34	42.5%
	Over 20 years	46	57.5%

Of the participants, 42.5% (n=34) were in the second year of the academic level followed by third- and fourth-year students with an equal percentage of 28.8% (n=23). Regarding the ages, 57.5% (n=46) of participants aged over 20 years, and 42.5% (n=34) of them were less than 20 years. The total scores of the participants in this study on the PNKAS indicated a satisfactory level of knowledge and attitudes regarding pediatric pain management at 86.3% (n=69) and an unsatisfactory level at 13.8% (n=11).

Table 2 shows the frequency and percentage of the top 10 correct answers. From the table, it can be identified that Q22 is the first top correct answer of undergraduate nursing students, where 69 of 80 students answered it with the correct answer with a percentage of 86.3% which was about pharma ecological management, and 85.9% (n=67) correctly answered regarding the assessment of pain. Moreover, 70% of participants (n=56) correctly responded to the non-pharmacological management.

**Table 2:** Frequency and percentage of top 10 correct answers of undergraduates nursing students The data has been presented as frequency N, (%)

Question number	Items	Key answer	Students’ answer
Q22	After the initial recommended dose of opioid analgesic, subsequent doses should be adjusted in accordance with the individual patient's response.	True	69 (86.3%)
Q8	Children who will require repeated painful procedures (i.e., daily blood draws), should receive maximum treatment for the pain and anxiety of the first procedure to minimize the development of anticipatory anxiety before subsequent procedures.	True	67(85.9%)
Q21	Anxiolytics, sedatives, and barbiturates are appropriate medications for the relief of pain during painful procedures.	False	57 (71.3%)
Q23	The child/adolescent should be advised to use non-drug techniques alone rather than concurrently with pain 3.	False	56 (70%)
Q25	In order to be effective, heat and cold should be applied directly to the painful area.	False	56 (70%)
Q24	Giving children/adolescents sterile water by injection (placebo) is Often a useful test to determine if the pain is real.	False	54 (67.5%)
14	Parents should not be present during painful procedures.	False	53 (66.3%)
20	Based on one's religious beliefs a child/adolescent may think that pain and suffering are necessary.	True	53 (66.3%)
Q10	Acetaminophen 650 mg PO is approximately equal in analgesic effect to codeine 32 mg PO.	True	47 (58.8%)
Q27	The recommended route of administration of opioid analgesics to children with brief, severe pain of sudden onset, e.g., trauma or postoperative pain, is	Intravenous	42 (52.5%)

Table 3 shows the frequency and percentage of the top 10 incorrect answers. From the table, it can be identified that Q37 is the first top incorrect answer of undergraduate nursing students, where 75 of 80 students answered it with the incorrect answer with a percentage of 93.8% provided to the "What do you think is the percentage of patients who over-report the amount of pain they have?" and 91.2% (n=73) incorrectly answered the questions regarding the occurrence of addiction. Moreover, 86.2% of the participants (n=69) incorrectly responded to the rout of administration in pharmacological management.

**Table 3:** Frequency and percentage of top 10 incorrect answers of undergraduates nursing students The data has been presented as frequency N, (%)

Question number	Items	Key answer	Students’ answer
Q37	What do you think is the percentage of patients who over-report the amount of pain they have? Circle the correct answer.	0 or 10%	75 (93.8%)
Q38	Narcotic/opioid addiction is defined as psychological dependence accompanied by overwhelming concern with obtaining and using narcotics for psychic effect, not for medical reasons. It may occur with or without the physiological changes of tolerance to analgesia and physical dependence (withdrawal). Using this definition, how likely is it that opioid addiction will occur as a result if treating pain with opioid analgesics? Circle the number closest to what you consider the correct answer.	1%	73 (91.2%)
Q7	Non-drug interventions (e.g., heat. music. imagery. etc.) are very effective for mild-moderate pain control but are rarely helpful for more severe pain.	False	70 (87.5%)
Q26	The recommended route of administration of opioid analgesics to children with prolonged cancer-related pain is	Oral	69 (86.2%)
Q31	A child with chronic cancer pain has been receiving daily opioid analgesics for two months. The doses increased during this time period. Yesterday the child was receiving morphine 20 mg/hour intravenously. Today he has been receiving 25 mg/hour intravenously for three hours. The likelihood of the child developing clinically significant respiratory depression is	Less than 1%	66 (82.5%)
Q39(A)	Patient A: Andrew is 15 years old and this is his first day following abdominal surgery. As you enter his room, he smiles at you and continues talking and joking with his visitor. Your assessment reveals the following information: BP = 120/80; HR = 80; R = 18; on a scale of 0 to 10 (0 = no pain/discomfort, 10 = worst pain/discomfort), he rates his pain as 8. A. On the patient’s record, you must mark his pain on the scale below. Circle the number that represents your assessment of Andrew’s pain.	8	66 (82.5%)
Q40(B)	Your assessment, above, is made two hours after he received morphine 2 mg IV. After he received the morphine, his pain ratings every half hour ranged from 6 to 8 and he had no clinically significant respiratory depression, sedation, or other untoward side effects. He has identified 2 as an acceptable level of pain relief. His physician’s order for analgesia is “morphine IV 1-3 mg q1h PRN pain relief.” Check the action you will take at this time:	Administer morphine 3 mg IV now	66 (80%)
Q39(B)	Your assessment, above, is made two hours after he received morphine 2 mg IV. After he received the morphine, his pain ratings every half hour ranged from 6 to 8 and he had no clinically significant respiratory depression, sedation, or other untoward side effects. He has identified 2 as an acceptable level of pain relief. His physician’s order for analgesia is “morphine IV 1-3 mg q1h PRN pain relief.” Check the action you will take at this time:	Administer morphine 3 mg IV now	65 (81.2%)
Q1	Observable changes in vital signs must be relied upon to verify a child's/adolescent's statement that he has severe pain.	False	63 (78.8%)
Q35	The most accurate judge of the intensity of the child’s/adolescent	The child/adolescent	53 (66.2%)

Additional statistical analysis for the relationship between demographic characteristics and the total score of the pediatric nursing knowledge and attitudes survey was performed as shown in Table 4. A T-independent samples test with age and a one-way ANOVA test with academic level were performed. There was no significant relationship between age and total score (t-value =-0.333; p-value=0.740). Similarly, the academic level among undergraduate nursing students did not show any significant relationship with the total score (f-value=0.936; p-value =0.397).

**Table 4:** Relationship between pediatric nursing knowledge and attitudes survey (PNKAS) and demographic characteristic Data are presented as N %, mean, mean ± SD, statistical significance at p-value<0.05

Age	N %	Mean	± Std.	t-value	p-value
Less than 20 years	34 (42.5%)	13.9118	2.56276	0.333	0.740
Over 20 years	46 (57.5%)	14.1304	3.13127		
Academic level	N %	Mean	± Std.	f-value	p-value
Second year	34 (42.5%)	13.9118	2.56276	0.936	0.397
Third year	23 (28.8%)	13.5652	3.23090		
Fourth year	23 (28.8%)	14.6957	2.99142		

## Discussion

Pain assessment and management present a significant challenge for healthcare providers due to its complex, subjective, and multifaceted nature, particularly in the pediatric population [19].

Pain assessment and management in pediatric patients is challenging for most of the health care providers. For effective pain management, nurses and physicians need to be aware of the effective assessment. The assessment, management, and evaluation of pain relief measures are necessary for positive patients’ outcomes in medical settings. Ineffective care, management, and diagnoses can cause an inadequate pain assessment and management that leads to prolongation of hospitalization. Untreated pain may cause significant disability and injuries. Nurses have a major and important part in children and infants' pain assessment and management.

The purpose of this study is to assess the nursing students’ knowledge and attitudes pertaining to children’s pain. The results of this study indicated that the majority of the study sample had a satisfactory level of knowledge and attitudes regarding pain assessments. Many factors lead to this satisfactory level of knowledge and attitudes toward pain management. For example, for the majority of the study sample, the completion of clinical training in pediatric areas leads to more knowledge and experiences. However, a descriptive study conducted in Turkey identified a lack of individual nursing care for patients concerning their comfort and education [20]. Thus, efforts should be made to improve nursing knowledge and practice regarding pain management.

The findings of the current study suggest no significant correlation between academic year and knowledge or attitudes regarding pediatric pain management. This aligns with research conducted at the University of Connecticut, USA, which reported no significant differences in knowledge and attitudes toward pediatric pain among nursing students [12]. Interestingly, the Connecticut study did find that junior students had a higher number of correct answers compared to seniors [12].

The current study revealed that a significant portion of incorrect answers pertained to pharmacological management, suggesting a knowledge gap in this area [12,14]. This could be attributed to two possible explanations: limited focus on pediatric applications or lack of knowledge retention by students.

These findings resonate with studies conducted in Egypt [14] and the United States [12], which reported similar weaknesses in pharmacological management knowledge among nursing students. In the Egyptian study, the lack of pharmacology coursework during the second year was identified as a contributing factor [14].

While the current study suggests a basic grasp of pain management principles, a Ghanaian study [11] found that students excelled in questions related to the subjective and multifaceted nature of pain experience. Conversely, an Egyptian study reported deficiencies in pain assessment among nursing students [14]. Additionally, a recent US study highlighted knowledge gaps among nursing faculty regarding pain management, with an average score of only 70 out of 100 on a pain knowledge and attitudes survey [21].

The current study findings indicate that students require improvement in pain assessment skills, pharmacological management, and knowledge related to medication addiction management. Interestingly, the study suggests students may possess theoretical knowledge of pain assessment but lack the practical experience to apply it effectively.

The study's findings suggest a need for strengthening educational programs for nursing students in pediatric pain management. Curriculum revisions should incorporate evidence-based strategies that effectively build knowledge and positive attitudes toward child pain assessment and management.

Beyond traditional lectures, incorporating diverse teaching methods can enhance student learning. Simulations, demonstrations with return demonstrations, and interactive case studies can foster deeper understanding and facilitate knowledge retention.

Furthermore, pharmacology education should emphasize the specific role nurses play in managing pediatric pain. Dedicating specific lectures to explore safe and effective drug interventions for children is crucial.

Finally, practical training is essential. Providing opportunities for nursing students to conduct pediatric pain assessments during hospital placements is highly recommended. Incorporating comprehensive training on specific pain assessment tools and their appropriate application with children would further equip future nurses to effectively address pediatric pain.

## Conclusions

The overall findings from this study indicate that undergraduate nursing students have satisfactory knowledge and attitudes regarding pediatric pain management. In addition, there is no significant relation between academic level and nursing students’ knowledge or attitudes regarding pediatric pain management. In addition, the knowledge deficiency is particularly related to the area of pain assessment and pharmacological pain management. Given the limited research on nursing knowledge and attitudes toward pediatric pain management in Saudi Arabia, this study highlights the need for enhanced education and training in this crucial area.

A small sample size was a limitation of this study. To gain a more comprehensive understanding of pediatric pain management knowledge and attitudes, future research should involve a larger and more diverse population. Including both pediatric nurses and undergraduate nursing students would enable valuable comparisons regarding their perceptions and preparedness.

## Appendices

**Table 5:** Knowledge and attitudes survey regarding pain true/false - circle the correct answer

Knowledge and attitudes survey regarding pain true/false - circle the correct answer		
T	F	1. Vital signs are always reliable indicators of the intensity of a patient’s pain.
T	F	2. Because their nervous system is underdeveloped, children under two years of age have decreased pain sensitivity and limited memory of painful experiences.
T	F	3. Patients who can be distracted from pain usually do not have severe pain. 4. Patients may sleep in spite of severe pain.
T	F	5. Aspirin and other nonsteroidal anti-inflammatory agents are NOT effective analgesics for painful bone metastases.
T	F	1. Vital signs are always reliable indicators of the intensity of a patient’s pain.
T	F	6. Respiratory depression rarely occurs in patients who have been receiving stable doses of opioids over a period of months.
T	F	7. Combining analgesics that work by different mechanisms (e.g., combining an NSAID with an opioid) may result in better pain control with fewer side effects than using a single analgesic agent.
T	F	8. The usual duration of analgesia of 1-2 mg morphine IV is 4-5 hours.
T	F	9. Research shows that promethazine (Phenergan) and hydroxyzine (Vistaril) are reliable potentiators of opioid analgesics.
T	F	10. Opioids should not be used in patients with a history of substance abuse.
T	F	11. Elderly patients cannot tolerate opioids for pain relief.
T	F	12. Patients should be encouraged to endure as much pain as possible before using an opioid.
T	F	13. Children less than 11 years old cannot reliably report pain so clinicians should rely solely on the parent’s assessment of the child’s pain intensity.
T	F	14. Patients’ spiritual beliefs may lead them to think pain and suffering are necessary.
T	F	15. After an initial dose of opioid analgesic is given, subsequent doses should be adjusted in accordance with the individual patient’s response.
T	F	16. Giving patients sterile water by injection (placebo) is a useful test to determine if the pain is real.
T	F	17. Vicodin (hydrocodone 5 mg + acetaminophen 500 mg) PO is approximately equal to 5-10 mg of morphine PO.
T	F	18. If the source of the patient’s pain is unknown, opioids should not be used during the pain evaluation period, as this could mask the ability to correctly diagnose the cause of pain.
T	F	19. Anticonvulsant drugs such as gabapentin (Neurontin) produce optimal pain relief after a single dose.
T	F	20. Benzodiazepines are not effective pain relievers unless the pain is due to muscle spasm.
T	F	21. Narcotic/opioid addiction is defined as a chronic neurobiologic disease, characterized by behaviors that include one or more of the following: impaired control over drug use, compulsive use, continued use despite harm, and craving.

**Table 6:** Multiple choice - place a check by the correct answer

Multiple choice - place a check by the correct answer
The recommended route of administration of opioid analgesics for patients with persistent cancer-related pain is _____ intravenous intramuscular subcutaneous oral rectal
The recommended route administration of opioid analgesics for patients with brief, severe pain of sudden onset such as trauma or postoperative pain is intravenous intramuscular subcutaneous oral rectal
Which of the following analgesic medications is considered the drug of choice for the treatment of prolonged moderate to severe pain for cancer patients? Codeine morphine meperidine tramadol
Which of the following IV doses of morphine administered over a 4 hour period would be equivalent to 30 mg of oral morphine given q 4 hours? Morphine 5 mg IV Morphine 10 mg IV Morphine 30 mg IV Morphine 60 mg IV
Analgesics for post-operative pain should initially be given around the clock on a fixed schedule only when the patient asks for the medication only when the nurse determines that the patient has moderate or greater discomfort
A patient with persistent cancer pain has been receiving daily opioid analgesics for 2 months. Yesterday the patient was receiving morphine 200 mg/hour intravenously. Today he has been receiving 250 mg/hour intravenously. The likelihood of the patient developing clinically significant respiratory depression in the absence of new comorbidity is less than 1% 1-10% 11-20% 21-40% e. > 41%
The most likely reason a patient with pain would request increased doses of pain medication is The patient is experiencing increased pain. The patient is experiencing increased anxiety or depression. The patient is requesting more staff attention. The patient’s requests are related to addiction.
Which of the following is useful for treatment of cancer pain? Ibuprofen (Motrin) Hydromorphone (Dilaudid) Gabapentin (Neurontin) All of the above
The most accurate judge of the intensity of the patient’s pain is a. the treating physician the patient’s primary nurse the patient the pharmacist the patient’s spouse or family
Which of the following describes the best approach for cultural considerations in caring for patients in pain: There are no longer cultural influences in the U.S. due to the diversity of the population. Cultural influences can be determined by an individual’s ethnicity (e.g., Asians are stoic, Italians are expressive, etc). Patients should be individually assessed to determine cultural influences. Cultural influences can be determined by an individual’s socioeconomic status (e.g., blue collar workers report more pain than white collar workers).
How likely is it that patients who develop pain already have an alcohol and/or drug abuse problem? < 1% 5 – 15% 25 - 50% 75 - 100%
The time to peak effect for morphine given IV is 15 min. 45 min. 1 hour 2 hours
The time to peak effect for morphine given orally is 5 min. 30 min. 1 – 2 hours 3 hours
Following abrupt discontinuation of an opioid, physical dependence is manifested by the following: sweating, yawning, diarrhea and agitation with patients when the opioid is abruptly discontinued Impaired control over drug use, compulsive use, and craving c. The need for higher doses to achieve the same effect. a and b
Case Studies Two patient case studies are presented. For each patient you are asked to make decisions about pain and medication. Directions: Please select one answer for each question. Patient A: Andrew is 25 years old and this is his first day following abdominal surgery. As you enter his room, he smiles at you and continues talking and joking with his visitor. Your assessment reveals the following information: BP = 120/80; HR = 80; R = 18; on a scale of 0 to 10 (0 = no pain/discomfort, 10 = worst pain/discomfort) he rates his pain as 8. A. On the patient’s record you must mark his pain on the scale below. Circle the number that represents your assessment of Andrew’s pain. 0 1 2 3 4 5 6 7 8 9 10 --------------------------------------------------------------------------------------------- No pain/discomfort Worst Pain/discomfort B. Your assessment, above, is made two hours after he received morphine 2 mg IV. Half hourly pain ratings following the injection ranged from 6 to 8 and he had no clinically significant respiratory depression, sedation, or other untoward side effects. He has identified 2/10 as an acceptable level of pain relief. His physician’s order for analgesia is “morphine IV 1-3 mg q1h PRN pain relief.” Check the action you will take at this time. Administer no morphine at this time. Administer morphine 1 mg IV now. Administer morphine 2 mg IV now. Administer morphine 3 mg IV now.
37. Patient B: Robert is 25 years old and this is his first day following abdominal surgery. As you enter his room, he is lying quietly in bed and grimaces as he turns in bed. Your assessment reveals the following information: BP = 120/80; HR = 80; R = 18; on a scale of 0 to 10 (0 = no pain/discomfort, 10 = worst pain/discomfort) he rates his pain as 8. A. On the patient’s record you must mark his pain on the scale below. Circle the number that represents your assessment of Robert’s pain: 0 1 2 3 4 5 6 7 8 9 10 --------------------------------------------------------------------------------------------- No pain/discomfort Worst Pain/discomfort B. Your assessment, above, is made two hours after he received morphine 2 mg IV. Half hourly pain ratings following the injection ranged from 6 to 8 and he had no clinically significant respiratory depression, sedation, or other untoward side effects. He has identified 2/10 as an acceptable level of pain relief. His physician’s order for analgesia is “morphine IV 1-3 mg q1h PRN pain relief.” Check the action you will take at this time: Administer no morphine at this time. Administer morphine 1 mg IV now. Administer morphine 2 mg IV now. Administer morphine 3 mg IV now.

## References

[1] Schellack N, Matimela M (2018). Paediatric pain management. S Afr Pharm J.

[2] Freund D, Bolick BN (2019). CE: assessing a child’s pain. Am J Nurs.

[3] Breau L, Camfield C, McGrath P (2007). Pain’s impact on adaptive functioning. J Intellect Disabil Res.

[4] Chiaretti A, Pierri F, Valentini P (2013). Current practice and recent advances in pediatric pain management. Eur Rev Med Pharmacol Sci.

[5] Kahsay H (2017). Assessment and treatment of pain in pediatric patients. Curr Pediatr Res.

[6] Zhu LM, Stinson J, Palozzi L (2012). Improvements in pain outcomes in a Canadian pediatric teaching hospital following implementation of a multifaceted, knowledge translation initiative. Pain Res Manag.

[7] Namnabati M, Abazari P, Talakoub S (2012). Identification of perceived barriers of pain management in Iranian children: a qualitative study. Int J Nurs Pract.

[8] Alsaqri SH (2018). Nursing student’s knowledge and attitudes toward pain management at Hail University, Saudi Arabia. Int J Adv Appl Sci.

[9] Liu YM, Lin GL, Chao KY, Jih HJ, Yang BH, Chiang YC (2020). Comparison of the effectiveness of teaching strategies for a pediatric pain management program for undergraduate nursing students: a quantitative evaluation using an objective structured clinical examination. Nurse Educ Pract.

[10] Li Y, Huang K, Cheng Y, Tong Y, Mo J (2019). Pain management by nurses in level 2 and level 3 hospitals in China. Pain Manag Nurs.

[11] Liu WN, Chang CF, Chung CH (2019). Clinical outcomes of bortezomib-based therapy in Taiwanese patients with multiple myeloma: a nationwide population-based study and a single-institute analysis. PLoS One.

[12] Laprise J (2016). Identification of student nurses’ knowledge and attitudes regarding pediatric pain management. University Scholar Projects.

[13] Al Omari O (2016). Knowledge and attitudes of Jordanian nursing students toward children’s pain assessment and management: a cross-sectional study. J Nurs Educ Pract.

[14] Gadallah MAEA, Hassan AM, Shargawy SAEH (2017). Undergraduate nursing students’ knowledge and attitude regarding pain management of children in upper Egypt. J Nurs Educ Pract.

[15] Stanley M, Pollard D (2013). Relationship between knowledge, attitudes, and self-efficacy of nurses in the management of pediatric pain. Pediatr Nurs.

[16] McCaa R (2017). Nurse perceptions of pain in pediatric traumatic brain injury: a pilot study. Pediatr Nurs.

[17] Lunsford L (2015). Knowledge and attitudes regarding pediatric pain in Mongolian nurses. Pain Manag Nurs.

[18] Manworren RC (2000). Pediatric nurses' knowledge and attitudes survey regarding pain. Pediatr Nurs.

[19] James SR, Nelson K, Ashwill J (2012). Nursing Care of Children-E-Book: Principles and Practice. https://www.zu.edu.jo/UploadFile/Library/E_Books/Files/LibraryFile_16954_18.pdf.

[20] Kol E, Arıkan F, İlaslan E (2018). A quality indicator for the evaluation of nursing care: determination of patient satisfaction and related factors at a university hospital in the Mediterranean Region in Turkey. Collegian.

[21] Duke G, Haas BK, Yarbrough S, Northam S (2013). Pain management knowledge and attitudes of baccalaureate nursing students and faculty. Pain Manag Nurs.

